# From Uterus to Brain: An Update on Epidemiology, Clinical Features, and Treatment of Brain Metastases From Gestational Trophoblastic Neoplasia

**DOI:** 10.3389/fonc.2022.859071

**Published:** 2022-04-13

**Authors:** Fulvio Borella, Stefano Cosma, Domenico Ferraioli, Mario Preti, Niccolò Gallio, Giorgio Valabrega, Giulia Scotto, Alessandro Rolfo, Isabella Castellano, Paola Cassoni, Luca Bertero, Chiara Benedetto

**Affiliations:** ^1^Division of Gynecology and Obstetrics 1, “City of Health and Science University Hospital”, University of Turin, Turin, Italy; ^2^Department of Surgical Sciences, University of Turin, Turin, Italy; ^3^Department of Oncology Surgery, Centre Léon Berard (CLB), Lyon, France; ^4^Department of Oncology, University of Torino, Torino, Italy; ^5^Pathology Unit, Department of Medical Sciences, “City of Health and Science University Hospital”, University of Turin, Turin, Italy

**Keywords:** gestational trophoblastic neoplasia, brain metastases (BMs), choriocarcinoma, chemotherapy, immunotherapy, treatment outcomes

## Abstract

In this review, we provide the state of the art about brain metastases (BMs) from gestational trophoblastic neoplasia (GTN), a rare condition. Data concerning the epidemiology, clinical presentation, innovations in therapeutic modalities, and outcomes of GTN BMs are comprehensively presented with particular attention to the role of radiotherapy, neurosurgery, and the most recent chemotherapy regimens. Good response rates have been achieved thanks to multi-agent chemotherapy, but brain involvement by GTNs entails significant risks for patients’ health since sudden and extensive intracranial hemorrhages are possible. Moreover, despite the evolution of treatment protocols, a small proportion of these patients ultimately develops a resistant disease. To tackle this unmet clinical need, immunotherapy has been recently proposed. The role of this novel option for this subset of patients as well as the achieved results so far are also discussed.

## Background

The term gestational trophoblastic disease (GTD) encompasses a spectrum of clinical-pathological disorders of abnormal placental development that include both non-malignant lesions and invasive tumors arising from placental villous and extravillous trophoblasts. In Europe, North and South America the estimated incidence of GTD is 1 per 500-1000 pregnancies, with an overall decline reported in recent years. However, in Asian women, the incidence of GTD remains significantly higher: 1 per 120 pregnancies (1–3). In this context, gestational trophoblastic neoplasia (GTN) is a group of rare tumors that includes invasive mole, choriocarcinoma, placental site trophoblastic tumors (PSTT), and epithelioid trophoblastic tumors (ETT). The incidence of choriocarcinoma is low and is estimated to 1–9 cases per 40,000 pregnancies, while PSTT and ETT account for 2–3% of all trophoblastic neoplasms (1). Occasionally non-pregnant and post-menopausal women may develop GTN (non-gestational choriocarcinoma). High-risk GTNs can develop distant metastases and the most common metastatic sites are lung (80%), vagina (30%), brain (10%), and liver (10%) (2–5). The purpose of this paper is to provide an overview and an update of the clinical-pathological features and treatments of GTN brain metastasis (BMs).

## Epidemiology of GTN Brain Metastasis

BMs represent the most common adult intracranial malignancy, and it is estimated that approximately 20–30% of adult patients with a solid neoplasm. will develop this complication during the course of their disease. However, data from autopsy studies performed on cancer patients showed a prevalence of solid BMs of up to 40% suggesting that many cases are asymptomatic and undiagnosed (6, 7). The most common solid tumors associated with the development of BMs are in order lung, breast, colorectal cancer, and melanoma, while in other tumor types, including gynecological ones, BMs are very rare (6–13). In this context, the prevalence of BMs from GTN is very low, although variations occurred over the years mainly due to the development of new diagnostic and therapeutic approaches. Historically, a large autopsy study performed in 1963 (14, 15) on 1096 patients with solid cancers identified 200 BMs and only one case derived from choriocarcinoma, while in 1968 an analysis of 393 autopsies of patients with BMs did not reveal any case from GTN. Autopsy studies performed in the 1980s (16, 17) and 1990s (18, 19) reported similar results, suggesting an extremely low prevalence of GTN origin in the general population of patients with BMs. Due to this rarity, the true prevalence of BMs in GTN patients is difficult to determine. In a literature review, Piura E (20). described the clinical-pathological features of 222 patients with BMs from GTN reported between 1980 and 2011, suggesting an overall prevalence of 11.4% with variations across the analyzed time periods. However, it should be noted that most of these data derive from case reports or very heterogeneous small series, thus overestimation of the prevalence rate is possible. Savage et al (21) suggest an incidence of BMs from GTN of 1 case per 22,000 molar pregnancies with brain involvement in about 20% of non-molar choriocarcinoma cases. These authors estimate a comprehensive risk of developing BMs from GTN of 2-3 cases per million pregnancies. Overall, based on the most significant case series published since 1980 we have observed a BMs prevalence ranging from 6.4% to 21.4% among the GTNs (**Table 1**) (21–35).

**Table 1:** Overview of clinical-pathological features, treatments, and outcomes of patients with BMs from GTN.

Author	Country	Time Period	BM (N)/Prevalence (%)	Previous Pregnancy	Median age at GTN diagnosis (yrs)	MeanInterval toBMs (mo)	Concomitant Metastatic site	Neurological symptoms(N, %)	Summary pf treatment regimen (N)	Survival
Weed 1980 (22)	USA	1966-1979	14	NA	20	NA	14 Lung 3 liver 2 vagina	Seizures (6, 42.8%) Headache (2, 14.3%) Vertigo (2, 14.3%)	14 MAC 13 RT	7 DOD 7 NED
Athanassiou* 1983 (23)	UK	1957-1981	69 (8.8%)	NA	28.5	14 (1-49) (available only for late BMs group)	NA	NA	MTX + other CHT agent (61) RT (14) MTX (8) Surgery (5)	OS: BMs presentation group: 38%; late BMs group: 0% (prior 1974) OS: BMs presentation group: 80%; late BMs group: 25% (after 1974)
Ishizuka 1983 (24)	Japan	1957-1980	36 (21.4%)	NA	NA	11 (0-47)	27 lung	Headache (14, 53.1%) Focal weakness (10, 37.5%) Disturbance of consciousness (7, 25.0%) convulsion (4, 1 5.6%), dizziness (4, 15.6%)	17 ActD 10 ActD + Surgery	26 DOD 1 AWD MS: 1.6 mo (0.1-50)
Liu 1983 (25)	China	1964-1978	34	NA	33.7	0.3-1	34 Lung 12 vagina	Headache (18, 52.9%), Focal weakness (10, 29.4%) Nausea/vomiting (10, 29.4%) Dizziness (6, 17.6%)	CHT (various regimens)	27 DOD 7 AWD
Rustin 1989 (26)	UK	1980-1988	25	9 mole 8 term 6 abortion 2 stillbirth	32	NA	23 lung	NA	25 EMA-CO RT (NA) Surgery (NA)	15 NED 7 DOD 2 AWD
Jones 1990 (27)	USA	1967-1987	19	9 mole 5 term 2 miscarriage 1 stillbirth 1 invasive mole 1 NA	32	11 (0-60)	18 lung 12 liver	Headache (9, 47.3%) Seizures (5, 26.3%) Dizziness (2, 10.5%)	18 MAC 19 RT 1 ActD 2 Surgery	14 DOD MS: 5.25 mo (0.1–24) 5 AWD MS 96 MO (48-180)
Evans 1995 (28)	USA	1966-1992	42/454 (9.3%)	16 mole 15 term 8 miscarriage 3 NA	NA	NA	NA	NA	35 MAC 4 ETP 42 RT 7 Surgery	19 DOD
Small 1996 (29)	USA	1962-1994	26/631 (4.1%)	12 term 7 mole 7 miscarriage	28	NA	NA	Hemiparesis (7, 27%) Headache (6, 23%) Asymptomatic (12, 46%)	26 RT 26 CHT 10 Surgery	14 DOD 10 NED 2 AWD
Schechter 1998 (30)	USA	1967-1994	21/242 (9%)	10 mole 5 miscarriage 3 term 1 stillbirth 1 none 1 NA	NA	NA	21 lung 8 liver	NA	21 RT 13 MTX +ActD 4 MTX 4 EMA-CO	13 DOD 7 NED 1 AWD
Suresh 2001 (31)	India	1991-1999	10	NA	NA	0	NA	NA	8 Surg 6 CHT	4 DOD 6 AWD
Cagayan 2005 (32)	Philippine	1992-2004	30/468 (6.4%)	60% mole 20% miscarriage 20% term	29.7	NA	21 lung 4 liver 7 none	Headache (20, 67%)	13 EMA-CO 6 MAC 5 EMA-CE 1 other 14 RT	Remission rate in presentation group: 35%; in late group 15%
Neubauer 2012 (33)	USA	1962-2009	11/162 (6.7%)	7 mole 3 term 1 miscarriage	31	NA	11 lung 3 liver	Headache (5, 45%) Asymptomatic (4, 36%) Vision changes (2, 18%) Tinnitus (2, 18%)	11 EMA-CO 11 RT 1 Surgery	6 NED 4 DOD 1 AWD
Savage 2015 (21)	UK	1991-2013	27	NA	32	<1 - 360	24 lung 4 liver 4 kidney	NA	24 EMA-CO 3 EMA-EP 19 EP previous definitive CHT 5 surgery 5 RT	23 NED 4 DOD
Xiao C 2015 (34)	China	1990-2013	109	41 mole 36 term 24 miscarriage	28	20 (1-288)	12 liver 10 kidney 4 liver + kidney 8 others	NA	109 FAEV + IT MTX 20 surgery 2 RT	OS: 71%
Gavanier 2019 (35)	France	1996-2016	22	13 term 7 miscarriage 1 mole	NA	NA	15 lung 3 liver 8 kidney 5 spleen 6 others	9 symptomatic (not specified) 6 asymptomatic	9 low dose EP 8 EMA-CO 2 IT MTX 1 not treated 1 surgery	OS rate: 69.8%
Xiao P 2021 (36)	China	2006-2020	14/612 (2.2%)	7 miscarriage 4 term 3 mole	32	25 (0.5-120)	14 lung 1 liver	4 headache (28%) 3 nausea/vomiting (21%) 1 vision changes (7%)	9 EMA-CO 9 IT MTX 4 RT	OS 78.57%
Li 2022 (37)	China	1990-2018	146 (the authors considered only 35 cases of patients undergoing craniotomy for this study)	19 term 9 miscarriage 7 mole	28	NA	24 none 7 kidney 1 liver and adrenal gland 1 intestine and vulva 1 bone 1 vagina	34 headache (97%) 34 vomiting (97%) 9 hemiplegia (26%) 7 loss of consciousness (20%) 6 convulsion (17%)	35 multi-agent CHT (not specified)	28 compete response (80%) 4 partial response (11.4%) 3 progression disease (8.6%)

*These authors divide the BMs patients in two clinical groups: those having BMs at initial disease presentation (BMs presentation group), and those who developed BMs during their treatment or who developed this complication after an initial complete or partial response (late BMs group).

AWD, alive with disease; BMs, brain metastasis; CHT, chemotherapy; DOD, died of disease; EMA-CE, etoposide, methotrexate, actinomycin D, cisplatin; EMA-CO etoposide, methotrexate, actinomycin D, cyclophosphamide, vincristine; EP, etoposide, cisplatin; FAEV, floxuridine, actinomycin D, etoposide, vincristine; GTN, gestational trophoblastic neoplasia; MAC, methotrexate, actinomycin D, cyclophosphamide; mo, months; NED, no evidence of disease; IT, intrathecal, MTX, methotrexate; NA, not available; OS, overall survival; RT, radiotherapy; yrs, years. Only relevant series published since 1980 and with at least 10 cases were reported.

## Clinical Presentation, Diagnosis, Staging, and Risk Stratification

The median age at diagnosis of BMs ranged from 20 to 33.7 years (**Table 1**). The interval time from previous pregnancy to BMs detection ranged from <1 to 360 months (**Table 1**), furthermore, some authors have distinctly considered patients who presented BMs at diagnosis versus those who developed brain disease during chemotherapy or after the end of treatment, as the latter had a worse prognosis (23, 32, 34).

Regarding the clinical presentation, overall, the symptoms of patients affected by BMs depend on the location, number, and size of the lesions, similarly to any lesion causing an intracranial mass effect. Mild or strong headache represents the most common presenting symptom occurring in up to 50% of multiple metastases or in the case of posterior fossa involvement. Up to 40% of patients show focal neurological deficits and 15-20% of cases develop seizures (8), however, BMs can also be found in asymptomatic patients (**Table 1**).

A specific manifestation of BMs from GTN is a suddenly and extensive intracranial hemorrhage associated or not with oncotic aneurysm because neoplastic cells derived from trophoblast retain the innate capacity to penetrate and erode blood vessels. In the literature, 30 cases of oncotic aneurysms secondary to BMs have been reported, most of them involving the middle cerebral artery and in 17% of cases more than 3 aneurysms were detected. The pathogenesis of an oncotic aneurysm is due to neoplastic vascular embolization with obstruction of distal cerebral vessels or with infiltration of the vasa vasorum followed by focal destruction of the intima, internal elastic lamina, and medial layers, leading to the development of an aneurysm. Brain angiography can be useful to study the characteristics of oncotic aneurysms, but identification of these lesions can be challenging in the case of cerebral hemorrhage (38, 39).

Brain computer tomography (CT) scan and/or magnetic resonance imaging (MRI) are the diagnostic choice to study cerebral lesions. As a general rule, brain MRI with or without intravenous gadolinium contrast is the gold standard for the assessment of BMs and shows a better sensitivity over contrast-enhanced CT for metastasis located in the posterior fossa where bone artifact can hide small metastases and/or leptomeningeal disease (8).

BMs from GTN may appear as single or multiple lesions and in most cases are located in the gray-white matter junction of the cerebral hemispheres (20, 34, 40). BMs are characterized by avid enhancement after administration of contrast due to the high vascularity of neoplastic tissue. BMs show high attenuation at non-enhanced computer tomography, and, at brain MRI, they are characterized by variable signal intensity depending on the chronicity of the intralesional hemorrhage (40).

Positron emission tomography (PET) does not play a relevant role in the diagnosis and staging of low-risk GTNs but it can be usefully associated with CT in patients with high-risk GTN and/or who have previously undergone multiple chemotherapy lines in order to monitor/detect primary and/or metastatic disease (41, 42). It should be noted that 18F‐fluorodeoxyglucose PET alone can have non-negligible false negative and positive results, so it is always advisable to associate this investigation with other diagnostic techniques (43).

BMs may be associated with other metastatic sites and in particular, brain involvement is often synchronous with lung metastasis (**Table 1**). Therefore in the case of detection of lung metastasis, a potential brain involvement should be considered and promptly investigated even without neurological symptoms (44). The extremely low incidence of BMs in post-molar pregnancy GTN would suggest that in the absence of neurological symptoms performing brain imaging is not indicated; however, for patients with non-molar choriocarcinoma, it is recommended to exclude brain involvement in all patients (21).

The detection of high serum and cerebrospinal fluid levels of β-hCG may also be helpful if a BM from GTN is suspected (45).

The staging system currently used for GTN has been developed by the FIGO (International Federation of Gynecology and Obstetrics) and includes four categories: 1) the tumor is limited to the uterus, 2) the tumor extends outside the uterus with local involvement of other genital structures (vagina, adnexa), 3) presence of lung metastases with or without local extra-uterine extension 4) brain, liver, kidneys and/or gastrointestinal tract involvement (46–48). In addition to tumor stage, several prognostic risk factors have been defined including patient’s age, antecedent pregnancy, interval months from index pregnancy, pretreatment serum hCG, largest tumor size, site and number of metastases and, the previous number of chemotherapy regimens. Women presenting a FIGO score of 0-6 should be considered low risk and a regimen with a single agent (methotrexate - MTX or dactinomycin) should be proposed. A high FIGO score (> 6) is predictive for chemoresistance in the case of single agent therapy, therefore combination chemotherapy should be used for these patients (46–48). Indeed, most patients with scores 0-4 are successfully treated with a single chemotherapy agent, while about 40% of women with a score of 5–6 need a polychemotherapy regimen after the first therapy (49). Patients with ultra high-risk (FIGO score ≥ 12) GTN have a 5-years OS rate of 67.9% and the presence of BMs is associated with a worse prognosis (RR 2.280, 95% CI 1.248–4.163, *p*-value = 0.007) (50).

## Treatment Options

Conversely to BMs derived by other types of tumors, which are predominantly treated with neurosurgical and radiation therapy approaches (8), in most cases BMs from GTNs are treated with cytotoxic chemotherapy (the most common protocols used are reported in **Table 2**). The proposed chemotherapy regimens have changed over the years, significantly improving the prognosis. Choriocarcinoma BMs series reported in the 1980s referring to cases from previous decades showed disappointing survival outcomes: Ishizuka et al (24) reported data regarding 27 patients treated with actinomycin D or actinomycin D plus neurosurgery resulting in 26 deaths, suggesting that this single-agent approach is not suitable. Probably, the poor efficacy of this drug lies in the difficulty of passing the blood-brain barrier (BBB). Furthermore, currently, actinomycin-D alone is indicated as a possible alternative to MTX in the treatment of low-risk, according to the FIGO score, GTN but not for high-risk GTN. Survival outcomes of 69 cases of choriocarcinoma BMs were reported in another series (23). These patients were mainly treated with systemic or intrathecal MTX alone or in combination with other unspecified chemotherapeutic agents. The authors reported worse survival outcomes in patients who developed BMs during treatment or after completing treatment compared to those who presented with BMs at first diagnosis, suggesting a progressive development of chemoresistance. Interestingly, they report an improvement in prognosis after 1974 for both the group that had early BMs diagnosis (38% vs 80%) and the group that developed BMs during or after treatment (0% vs 25%). These survival improvements were explained thanks to i) use of MTX in patients with lung metastases (often concomitant with brain metastases); ii) early detection of central nervous system lesions and iii) use of A combination of systemic and intrathecal therapy. Liu et al (25) reported the outcomes of 34 patients with metastatic brain disease treated with heterogeneous regimens mainly based on 6-mercaptopurine, 5-fluorouracil, and MTX also suggesting a complementary role of unspecified Chinese herbs, but most of the patients of this series died less than a month after diagnosis. Savage et al (21) reported a good overall survival (OS) rate (85%) treating most patients with EMA-CO (**Table 2**) preceded by low dose etoposide and cisplatin. Interestingly, none of these patients was subjected to whole-brain radiation therapy, and only 5 cases required brain stereotactic radiotherapy (RT) for residual tumor at the end of chemotherapy. Xiao et al (34) reported the outcomes of 109 GTN patients with BMs using a combination of systemic chemotherapy (FAEV regimen, **Table 2**) (51) plus intrathecal injection of MTX. Also in this series, only 2 patients received brain RT. The 5-year OS was 71.1%, rising to 85.5% for patients receiving primary chemotherapy in dedicated referral GTN centers. The authors also report as independent poor prognostic indicators for survival: FIGO score > 12 (Hazard ratio-HR 1.279, 95% CI 1.061-1.541, p-value = 0.010), failure of previous multidrug chemotherapy (HR 3.177, 95% CI 1.277-7.908, *p*-value = 0.013), and concomitant renal involvement (HR: 2.654, 95% CI 1.125-6.261, p-value = 0.026). The use of MTX-based intrathecal therapy is controversial: an early and intensive therapy with high doses of MTX can induce sudden necrosis of the BM and cause cerebral hemorrhage[ (23). Response rates of over 80% were achieved when intrathecal MTX was used alongside EMA-CO or EMA-EP (21, 26), however, a recent study reported a higher response rate in patients who underwent systemic chemotherapy without intrathecal MTX, therefore, the role of this therapeutic approach remains to be defined (35). The proposed regimen of intrathecal MTX is reported in **Table 2** (52).

**Table 2:** most common multidrug regimens used for treating BMs from GTN.

	Dose	Administered on day(s)	Route of administration
**EMA-CO protocol** (21) every 21 days			
Dactinomycin	0.5 mg	Week 1, day 1 and day 2	IV
Etoposide	100 mg/m^2^	Week 1, day 1 and day 2	IV
MTX	1000 mg/m^2^	Week 1, day 1	IV > 12 h
Folinic acid	30 mg	Week 1, Day 1	Oral, every 6 h x 12 doses, 32 h after start MTX
Vincristine	0.8 mg/m^2^	Week 2, Day 8	IV
Cyclophosphamide	600 mg/m^2^	Week 2, Day 8	IV
**EMA-EP protocol** (21) every 21 days			
Dactinomycin	0.5 mg	Week 1, day 1 and day 2	IV
Etoposide	100 mg/m^2^	Week 1, day 1 and day 2	IV
MTX	1000 mg/m^2^	Week 1, day 1	IV > 12 h
Folinic acid	30 mg	Week 1, Day 1	Oral, every 6 h x 12 doses, 32 h after start MTX
Etoposide	150 mg/m^2^	Week 2, Day 8	IV
Cisplatin	75 mg/m^2^	Week 2, Day 8	IV
**FAEV protocol** every 21 days ls until normalization of β-hCG level (51)			
Vincristine	2 mg	Day 1	IV, bolus 3 h before dactinomycin infusion
Dactinomycin	200 μg/m2	Day1-5	IV, Infusion duration > 30 min
Etoposide	100 mg/m2	Day1-5	IV, Infusion duration > 30 min
Floxuridine	800 mg/m2	Day1-5	IV, Infusion duration > 8 h
**Intratechal MTX (concomitant with EMA-CO/EP)** (52)			
MTX	12.5 mg	Day 8	Intrathecal
Folinic acid	15 mg	Day 8	IM, 24 and 36 h after intrathecal MTX

BMs, brain metastasis; EMA-CO etoposide, methotrexate, actinomycin D, cyclophosphamide, vincristine; EMA-EP etoposide, methotrexate, actinomycin D, etoposide, cisplatin; FAEV, floxuridine, actinomycin D, etoposide, vincristine; GTN, gestational trophoblastic neoplasia; IM, intramuscular; IV, intravenous; MTX, methotrexate.

As previously mentioned, the role of RT has progressively lost its importance in the treatment of GTN’s BMs. In 1980 Weed (22) et al. reported the survival outcomes of 14 patients affected by BMs from GTN. Of them, 13 were treated with whole brain RT plus systemic chemotherapy, but only 7 (50%) achieved a complete response. Athanassiou et al (23) also reported the survival outcomes of a series of patients treated with whole-brain RT: 6/6 patients who developed late BMs died, while out of 8 patients who developed early BMs only 2 survived. Other authors (26, 27, 32) also reported an overall poor efficacy of RT, while Schechter et al (30) reported a survival advantage in a small cohort of patients (N = 10) who received a dose greater than 2200 cGy compared to those who were subjected to lower doses (N = 11). Interestingly, the results of a recent study carried out at the French Trophoblastic Disease Reference Centre on 21 patients with BMs from GTN treated with multidrug chemotherapy without concomitant whole-brain RT showed a higher OS rate of 81.5% (excluding 3 cases of early death due to intracranial hemorrhage) compared with OS rates of 75% from studies combining whole-brain RT with multidrug chemotherapy (35). Based on these results, the authors of this study do not support the role of whole-brain RT in patients with BMs from GTN at initial diagnosis (35).

Furthermore, experiences in the treatment of BMs from other neoplasms suggest a decline in neurocognitive function (53, 54) and due to the mean young age of patients affected by BMs from GTN, this issue should also be taken into consideration.

The potential neurocognitive damage in patients with BM’s from GTN undergoing brain RT is poorly reported in the literature. Athanassiou et al (23) reported several side effects in patients who underwent whole-brain RT (N=14) for BMs from GTN including hemianopia, hemiparesis, epilepsy, episodes of amnesia, headache, aphasia, and cognitive deficits.

Recently, data from 35 patients treated with multi-agent chemotherapy regimens and craniotomy showed an OS of 80%, however, in 26 patients craniotomy was performed in critical condition for intracranial hemorrhage and hypertension (37).

The role of intracranial surgery is actually limited to salvage treatment in case of intracranial hypertension, hemorrhage, edema, or for resection of chemoresistant metastasis (23, 28, 38).

## Immunotherapy

The introduction of immune checkpoint inhibitors (ICIs) constitutes a paradigm shift in cancer treatment, in particular for melanoma, non-small cell lung cancer, and renal cell cancer (55–59), but in other contexts, such as in the treatment of gynecological neoplasms their role is controversial and ICIs have been approved for treating only some specific subtypes of tumors (60–65).

The main ICIs targets include programmed cell death 1 (PD-1), programmed cell death 1 ligand 1 (PD-L1), and cytotoxic T lymphocyte antigen 4 (CTLA-4). PD-1 is a cell surface co-inhibitory receptor member of the CD28/CTLA-4 family. Normally, it is expressed on lymphocytes but it can also be found on other immune cells including monocytes and natural killer T cells. Following its activation, PD-1 receptor promotes the production of pro-inflammatory molecules such as interferon-γ (IFN-γ), tumor necrosis factor-α (TNF- α), and interleukin2 (IL-2), inhibits CD8+ T cell proliferation and activation and suppresses the activity of regulatory T lymphocytes. PD-1 may also target tumor cells and tumor-associated macrophages helping to maintain an immunosuppressive tumor environment (66, 67). CTLA-4 is another fundamental T cell protein associated with immune regulation. CTLA-4 is expressed by both CD4+ and CD8+ T cells and exerts its immunosuppressive effects through the modulation of regulatory T cells (68). The inhibition of the binding between PD-1 and CTLA-4 and their receptors by ICIs may improve the cytotoxic CD8+ T-cell effectiveness eliciting a higher antineoplastic effect.

Historically, already in the 1960s, immunotherapeutic approaches were used for the treatment of BMs from GTN, for example through skin graft from the patient’s husband with the rationale of stimulating immunity against paternal antigens or promoting nonspecific immunological stimuli through the Bacillus of Calmette-Guerin, but these methods were soon abandoned (23).

In recent years, the treatment of chemoresistant GTNs through ICIs has gained significant interest and has been actively investigated, even in patients with BMs. Zong et al (69) investigated the expression of molecules belonging to B7 family check-point proteins in 112 GTN: 68 choriocarcinomas, 33 PSTT.and 11 ETT. PD-L1 and B7-H3 were both highly expressed in all tumors, while PD-L2 was found in 87.5% of the samples. PD-L1, B7-H3, and VISTA expression were significantly higher in choriocarcinomas and PSTTs compared with ETTs. High expression of PD-L1 was also observed in GTNs in previous studies (70–72). These results suggest a strong rationale for the use of immunotherapy in these tumors.

Encouraging results were obtained with the use of ICIs in the TROPHIMMUN Phase II Trial (73). This study included 15 patients who experienced progression disease from GTN and were treated with Avelumab (anti PD-L1 drug) 10 mg/kg intravenously every 2 weeks on monotherapy achieving a complete response in approximately 50% of patients. Unfortunately, this study considered the presence of BMs as an exclusion criterion. Furthermore, Ghorani et al (74) reported the outcomes of 4 patients with drug-resistant GTN treated with pembrolizumab, an anti PD-1 antibody. Of these 4 patients, 3 achieved a complete response to pembrolizumab, including one patient with metastatic brain disease. Good clinical results with pembrolizumab were also obtained in a 30-year-old patient with multiple GTN metastasis including within the central nervous system, refractory to classical chemotherapy regimens (75). Unfortunately, given the rarity of BMs from GTN, there are no trials on the specific use of ICIs for this disease.

## Conclusions

GTN BMs remain a rare occurrence often associated with other metastatic sites and harboring a poorer prognosis, especially because of the complications such as rupture of an oncotic aneurysm. An immediate multidisciplinary approach is recognized as the best strategy for patient assessment and decision-making regarding the initial treatment of NTG with brain metastases. The multidisciplinary team should include a neuroradiologist, a neurosurgeon, and a radiotherapist led by a specialist with knowledge of all treatment modalities involved in the management of GTN (44).

Despite the rarity of this complication, excellent therapeutic results have been obtained through a progressive refinement of multi-agent chemotherapy regimens, achieving complete remissions in many cases. Nevertheless, some patients develop chemoresistant disease. In this subset of patients, some further progress has been recently made thanks to the use of ICIs, but further studies are needed to confirm the role of immunotherapy in GTN BMs resistant to classical chemotherapy regimens.

## Author Contributions

Conceptualization: FB, SC; Methodology: FB, SC; Resources: CB; Literature research: DF, MP, GV, GS, NG, AR; Data curation, FB, SC, DF, LB; Interpretation of data: all authors. Writing—original draft preparation: FB, SC, DF, LB; Writing—review and editing: all authors; Supervision: CB; Project administration: FB, SC, CB; All authors have read and agreed to the published version of the manuscript.

## Conflict of Interest

GV has received personal fees from Roche, AstraZeneca, Tesaro, PharmaMar and Amgen.

The remaining authors declare that the research was conducted in the absence of any commercial or financial relationships that could be construed as a potential conflict of interest.

## Publisher’s Note

All claims expressed in this article are solely those of the authors and do not necessarily represent those of their affiliated organizations, or those of the publisher, the editors and the reviewers. Any product that may be evaluated in this article, or claim that may be made by its manufacturer, is not guaranteed or endorsed by the publisher.
